# Pharmacological inhibition of TAK1, with the selective inhibitor takinib, alleviates clinical manifestation of arthritis in CIA mice

**DOI:** 10.1186/s13075-019-2073-x

**Published:** 2019-12-17

**Authors:** Scott A. Scarneo, Liesl S. Eibschutz, Phillip J. Bendele, Kelly W. Yang, Juliane Totzke, Philip Hughes, David A. Fox, Timothy A. J. Haystead

**Affiliations:** 1 Department of Pharmacology and Cancer Biology, Duke University School of Medicine, LSRC C112, 308 Research Drive, Durham, NC 27710 USA; 2 Bolder BioPATH, Inc., 5541 Central Ave., Suite 160, Boulder, CO 80301 USA; 3 Division of Rheumatology and Clinical Autoimmunity Center of Excellence, University of Michigan, Ann Arbor, MI 48109 USA

**Keywords:** Inflammatory arthritis, TAK1, Inflammation, Kinases, Small molecule inhibitor, Therapeutics

## Abstract

**Objectives:**

To examine the ability of takinib, a selective transforming growth factor beta-activated kinase 1 (TAK1) inhibitor, to reduce the severity of murine type II collagen-induced arthritis (CIA), and to affect function of synovial cells.

**Methods:**

Following the induction of CIA, mice were treated daily with takinib (50 mg/kg) and clinical scores assessed. Thirty-six days post-CIA induction, histology was performed on various joints of treated and vehicle-treated animals. Inflammation, pannus, cartilage damage, bone resorption, and periosteal bone formation were quantified. Furthermore, pharmacokinetics of takinib were evaluated by LC-MS in various tissues. Rheumatoid arthritis fibroblast-like synoviocytes (RA-FLS) cells were cultured with 10 μM takinib and cytokine secretion analyzed by cytokine/chemokine proteome array. Cytotoxicity of takinib for RA-FLS was measured with 24 to 48 h cultures in the presence or absence of tumor necrosis factor (TNF).

**Results:**

Here, we show takinib’s ability to reduce the clinical score in the CIA mouse model of rheumatoid arthritis (RA) (*p* < 0.001). TAK1 inhibition reduced inflammation (*p* < 0.01), cartilage damage (*p* < 0.01), pannus, bone resorption, and periosteal bone formation and periosteal bone width in all joints of treated mice compared to vehicle treated. Significant reduction of inflammation (*p* < 0.004) and cartilage damage (*p* < 0.004) were observed in the knees of diseased treated animals, with moderate reduction seen in the forepaws and hind paws. Furthermore, the pharmacokinetics of takinib show rapid plasma clearance (t_½_ = 21 min). In stimulated RA-FLS cells, takinib reduced GROα, G-CSF, and ICAM-1 pro-inflammatory cytokine signaling.

**Conclusion:**

Our findings support the hypothesis that TAK1 targeted therapy represents a novel therapeutic axis to treat RA and other inflammatory diseases.

## Introduction

Rheumatoid arthritis (RA) is a chronic inflammatory disease in which hyperactivated immune cells induce maladaptive persistent inflammation in the joints leading to synovial inflammation and bone remodeling. RA currently affects roughly 1% of people in the USA [1, 2]. The pathogenesis of RA is tightly linked to inflammation [3–7]. Acute increases in pro-inflammatory cytokines confer survival advantage by promoting immune responses that limit tissue damage and initiate tissue repair following injury or pathogen exposure [8–10]. However, sustained elevations in pro-inflammatory cytokines elicit chronic tissue damage and pain which are maladaptive, significantly impairing a patient’s lifestyle. Tumor necrosis factor (TNF; formerly denoted TNFα [11]) has been shown to play an integral role in the regulation of pro-inflammatory signaling, especially in the affected joints of RA patients. Therapies such as anti-TNF antibodies have been used as a means to mitigate the chronic pro-inflammatory milieu associated with RA.

Currently, TNF-sequestering antibodies or soluble TNF receptors are indicated for the treatment of rheumatoid arthritis and Crohn’s disease [12, 13]. However, up to 40% of patients fail to respond to anti-TNF biologics, which is often attributed to immune sensitization to the therapeutic agent [14]. Additionally, anti-TNF treatments require intravenous infusions or regular self-injection, leading to greater noncompliance rates. Identification of critical TNF signaling kinases such as transforming growth factor beta-activated kinase 1 (TAK1) represents a novel means to target TNF-induced inflammation with a small molecule inhibitor which may be formulated as an orally available treatment. A key signaling element in the TNF pro-survival/inflammatory response pathway is the protein kinase TAK1. TAK1 plays a crucial role in mediating activation of protein kinase-mediated signaling pathways implicated in the pathogenesis of inflammatory and oncogenic processes, such as nuclear factor kappa-light chain enhancer of activated B cells (NF-κB) and mitogen-activated protein kinases (MAPKs) [15, 16]. Because of its critical role in these pathways, TAK1 has emerged as a potential therapeutic target for the treatment of inflammatory-mediated diseases like RA, ankylosing spondylitis, and inflammatory bowel disease [17–19]. Small molecule drugs that selectively target TAK1 may represent a safer and more effective option for long-term management of chronic inflammation. Our recent discovery of the takinib scaffold has identified a highly specific potent inhibitor of TAK1 (~ 9 nM), and we hypothesize that this presents an approach to regulating TNF production and signaling [20, 21].

In this study, we use the well-established type II collagen-induced arthritis (CIA) mouse model, which engenders symptoms which mimic human disease to evaluate the putative therapeutic effect of TAK1 inhibition on RA in vivo [22, 23]. We show that takinib significantly reduced the clinical arthritic score of CIA mice and mitigated rescued CIA-induced weight loss. Furthermore, takinib significantly reduced inflammation, cartilage damage, pannus formation, and bone resorption. Evaluation of the individual joints showed the greatest influence of takinib in the knee joints, with reduction of inflammation, pannus, bone resorption, and cartilage damage seen in all takinib-treated groups. Furthermore, pharmacokinetics (PK) of takinib showed rapid blood serum clearance with a t_1/2_ of 21 min in serum. Overall, these results demonstrate the potential for TAK1 as a therapeutic target to control inflammatory signatures of RA and reduce disease burden.

## Materials and methods

### Animal care and use statement

The DBA/1 CIA model was conducted in accordance with The Guide for the Care & Use of Laboratory Animals (8th Edition) and therefore in accordance with all Bolder BioPATH IACUC approved policies and procedures. The Bolder Biopath IACUC approved a “blanket” IACUC protocol for this specific working protocol (BBP-001). No acceptable alternative test systems were identified for the animals used in this study. All studies were performed in male DBA/1 mice, and animals which failed to develop CIA arthritis by day 21 were excluded from analysis. Clinical evaluation was performed under experimenter blinded conditions.

Pharmacokinetic studies were approved and carried out in accordance with the University of North Carolina-Chapel Hill, Institution Animal Care and Use Committee (IACUC), and conformed to the National Institutes of Health Guide for the Care and Use of Laboratory Animals. Mice were housed in a temperature- and humidity-controlled facility under 12-h light/dark cycle (lights on at 7 am) with access to food and water ad libitum.

### Collagen type II-induced arthritis (CIA) induction

Collagen was prepared as a 4 mg/ml solution in 0.01 N acetic acid. Equal volumes of 4 mg/ml collagen and 5 mg/ml Freund’s complete adjuvant were emulsified by hand mixing with syringes for approximately 5 min, at which point a bead of this material holds its form when placed in water. On study days 0 and 21, animals were anesthetized with isoflurane and given intradermal injections of a total of 400 μg of type II collagen in Freund’s complete adjuvant at the base of the tail [24, 25].

### Experimental design

Mice were randomized by body weight into treatment groups on study day 18. Treatment was initiated following enrollment. On study day 36, the mice were euthanized for necropsy. Clinical scores were given for each of the paws (right front, left front, right rear, left rear) on study days 18–36. Experimenter was blinded from the treatment group during clinical evaluation and scoring.

### Immunohistological staining

After 1–2 days in fixative and 4–5 days in 5% formic acid for decalcification, tissues were trimmed and processed for paraffin embedding. Paws were embedded in paraffin in the frontal plane, and the knees were embedded with the patella facing down. Ankles, if left attached to the hind paw, were also embedded in the frontal plane but may be detached and sectioned in the sagittal plane for special purposes. Left/right pairs were typically embedded in the same block. Sections were cut and stained with toluidine blue.

### Scores for synovitis, pannus formation, degradation of cartilage, and bone

#### Paw score criteria

The paw score criteria were as follows: 0 = Normal. 0.5 = Very minimal, affects only 1 joint or minimal multifocal periarticular infiltration of inflammatory cells. 1 = Minimal infiltration of inflammatory cells in synovium and periarticular tissue of affected joints. 2 = Mild infiltration of inflammatory cells. When referring to paws, generally restricted to affected joints (1–3 affected). 3 = Moderate infiltration with moderate edema. When referring to paws, restricted to affected joints, generally 3–4 joints and the wrist or ankle. 4 = Marked infiltration affecting most areas with marked edema, 1 or 2 unaffected joints may be present. 5 = Severe diffuse infiltration with severe edema affecting all joints (to some extent) and periarticular tissues.

#### Knee score criteria

The knee score criteria were as follows: 0 = Normal. 0.5 = Very minimal, affects only one area of the synovium or minimal multifocal periarticular infiltration of inflammatory cells. 1 = Minimal infiltration of inflammatory cells in synovium and periarticular tissue of affected synovial areas. 2 = Mild diffuse infiltration of inflammatory cells. 3 = Moderate diffuse infiltration of inflammatory cells. 4 = Marked diffuse infiltration of inflammatory cells. 5 = Severe diffuse infiltration of inflammatory cells.

#### Cartilage damage score criteria

The cartilage damage score criteria were as follows: 0 = Normal. 0.5 = Very minimal = Affects marginal zones only of one to several areas (knees) or joints (paws). 1 = Minimal = Generally minimal to mild loss of toluidine blue staining (proteoglycan) with no obvious chondrocyte loss or collagen disruption in affected joints/areas. 2 = Mild = Generally mild loss of toluidine blue staining (proteoglycan) with focal areas of chondrocyte loss and/or collagen disruption in some affected joints/areas. Paws may have one or two digit joints with near total to total loss of cartilage. 3 = Moderate = Generally moderate loss of toluidine blue staining (proteoglycan) with multifocal chondrocyte loss and/or collagen disruption in affected joints/areas. Paws may have three or four joints with near total or total loss. In the knee, some matrix remains on any affected surface with areas of severe matrix loss. 4 = Marked = Marked loss of toluidine blue staining (proteoglycan) with multifocal marked (depth to deep zone or tidemark) chondrocyte loss and/or collagen disruption in most joints with a few unaffected or mildly affected. In the knee, one surface with total to near total cartilage loss. 5 = Severe = Severe diffuse loss of toluidine blue staining (proteoglycan) with severe (depth to tide mark) chondrocyte loss and/or collagen disruption in most or all joints.

### Pharmacokinetics

C3-tag mice were injected intraperitoneally (i.p.) with 50/75mpk takinib and sacrificed at 0, 1 h, 2 h, 4 h, 8 h, 16 h, and 24 h. At the indicated time points, the heart, spleen, kidney, liver, tumor, blood, plasma, muscle, and lung were harvested. All samples were frozen and stored at − 80 °C. Before quantifying the takinib in serum, a standard curve was constructed using HS-219, a close structural analog of takinib that serves as an internal standard. This internal standard solution was used for tissue homogenization. LC-MS analysis was performed at the Duke Proteomics and Metabolomics Core Facility [26]. The final concentration of takinib in the plasma was calculated per milliliter of plasma.

### Cell culture

Rheumatoid arthritis fibroblast-like synoviocytes (RA-FLS) cells were prepared from surgical synovial samples at the University of Michigan as previously described [27]. Cells were isolated from 4 individual patients (3 female, 1 male) and passaged no more than 2 times before use. Cells were cultured in CRML media, 10% FBS, 1% penicillin-streptomycin (PS), and 1% glutamate.

### Cytokine/chemokine proteome profile

RA-FLS cells were activated by addition of LPS 10 ng/mL in the presence or absence of 10 μM takinib or DMSO. Twenty-four hours after treatment, supernatant was added to Human Cytokine proteome array (R&D Systems). Cytokine kit was conducted in accordance with manufacturer protocol. Chemiluminescence was used to visualize protein quantities.

### Kinase proteome profiling

Briefly, 10^6^ cells were plated and either no treatment (naïve) or pretreated with takinib (10 μM) or vehicle (DMSO) for 30 min prior to stimulation with TNF (30 ng/mL) for 30 min at 37 °C in 5% CO_2_ for phospho-kinase assay (R&D Systems). NF-κB and cytokine assays were performed as previously described; however, cells were incubated for 24 h post-TNF (30 ng/mL) or LPS (10 ng/mL) stimulation.

### Cell viability assay

RA-FLS cells were cultured and treated as previously described. Briefly, cells were plated at 80% confluency ~ 10^4^ in 96-well plates and treated with either takinib at various concentrations or takinib + TNF (30 ng/mL) for 24 or 48 h and compared to vehicle-treated samples. Cell death was quantified using Cell Titer Glo 2.0 (Promega) according to the manufacturer’s protocol.

### Quantification and statistical analysis

**Fig. 1:**
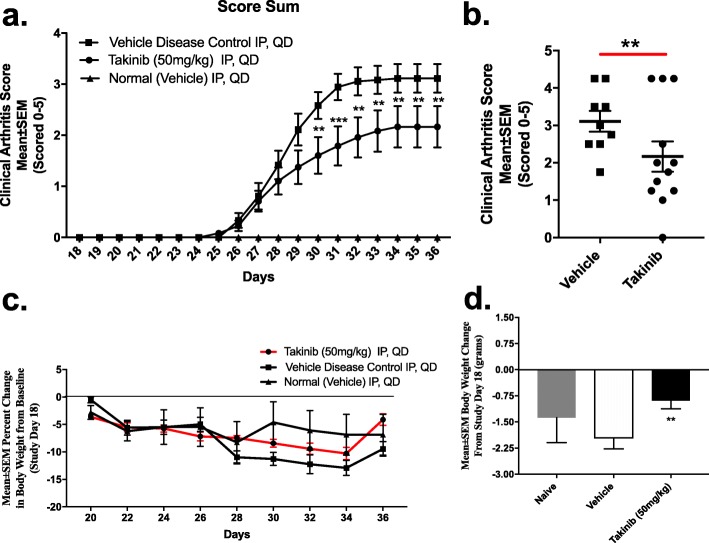
Takinib reduces the clinical arthritis score. DBA/1 mice disease induced with CIA and demonstrated clinical arthritic scores around day 25, which persisted throughout the study. Mice were treated daily with vehicle control or takinib (50 mg/kg) intraperitoneal injections. Takinib-treated mice show a reduction in clinical arthritic score compared to vehicle control (**a**). Day 36 clinical score between takinib and vehicle treated (**b**). Weight change over 2 weeks of treatment (**c**). Overall weight change between treatment groups (**d**). *n* = 4 ± SEM/normal (vehicle) group, *n* = 9–12 ± SEM/vehicle disease control and takinib groups. *p < 0.05, **p < 0.01, ***p < 0.001 two-way ANOVA, Dunnett’s post hoc

**Fig. 2:**
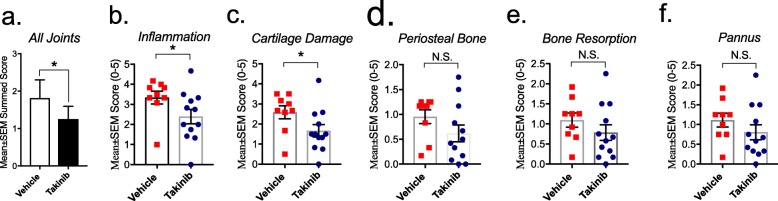
Takinib reduced the histological scores of CIA mice. Overall, takinib significantly reduced all joints summed scores compared to vehicle control (**a**). Reduction of inflammation (I), pannus (P), cartilage damage (CD), bone resorption (BR), and periosteal bone formation (PB) were seen in all joints of vehicle- and takinib-treated animals (**b**–**f**). *n* = 4 ± SEM/normal (vehicle) group, *n* = 9–12 ± SEM/vehicle disease control and takinib groups. *p < 0.05, **p < 0.01, ***p < 0.001 two-way ANOVA, Dunnett’s post hoc

**Fig. 3:**
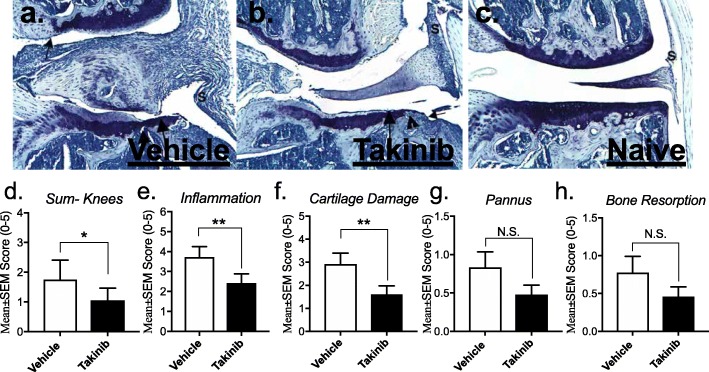
Takinib treatment reduces individual joint scores in knees. Knee from a vehicle control animal has marked inflammation (S) and moderate cartilage damage (large arrow) with minimal pannus (small arrow) and bone resorption (arrowhead) (**a**). Knee from an arthritic animal treated with 50 mg/kg of takinib has moderate inflammation (S) and cartilage damage (large arrow) with minimal pannus (small arrow) and bone resorption (arrowhead), as well as very minimal periosteal bone formation (not pictured) (**b**). Knee from a normal control animal has no lesions. S identifies synovium (**c**). Reduction of sum knees, inflammation (I), pannus (P), cartilage damage (CD), and bone resorption (BR) were seen in the knees of vehicle- and takinib-treated animals (**d**–**h**). *n* = 4 ± SEM/normal (vehicle) group, *n* = 9–12 ± SEM/vehicle disease control and takinib groups. *p < 0.05, **p < 0.01, ***p < 0.001 two-way ANOVA, Dunnett’s post hoc

GraphPad Prism 7 was used for statistical analysis of clinical score comparisons, histological analysis, and plasma concentrations. Two-way ANOVA followed by Dunnett’s multiple comparison test was performed for mean clinical score sum (Fig. 1); weight change over time (Fig. 1); joint score sum for all joints, knees, and forepaw and hind paw (Figs. 2, 3); protein analysis (Figs. 5, 6); and cell survival (Fig. 6g). A linear regression of LC-MS data on takinib was used for PK studies. T_½_ was determined by non-linear one-phase decay curves. Student *T* tests were performed for periosteal bone width (Additional file 1: Figure S2) and cell survival (Fig. 6f, g). For each analysis, total *n* and SEM are presented in the figure legend. An alpha of 0.05 was used for all statistical analysis.

## Results

### Takinib reduces the clinical score of CIA RA mice

TAK1 inhibition with takinib reduces clinical RA score in a CIA RA mouse model. All animal studies were performed by Bolder BioPath (Boulder, CO) following industry standard procedures for the CIA mouse model. Disease development was monitored throughout the study. Due to a variability in disease uptake from the CIA model of inflammatory arthritis, we performed an outlier test to determine mice which failed to develop disease; this was determined by mice which fell further than 2 standard deviations (SD) from the mean. Animals identified from the outlier test (e.g., 3/12 mice in vehicle control failed to develop symptoms) were excluded from data analysis (Additional file 1: Table S1). Vehicle disease control mice increased through study day 34 and then maintained at that level through study termination. Mean daily clinical arthritis scores in mice treated with takinib significantly differed on days 30–36 from vehicle disease control (Fig. 1a). Furthermore, at day 36, some of the takinib-treated mice had less severe disease than any mice in the vehicle-treated group (Fig. 1b). When comparing the area under the curve (AUC) between takinib and vehicle, takinib reduced AUC by 32% from vehicle control (Additional file 1: Table S1). Takinib attenuated body weight reduction compared to vehicle control, with 50% less overall body weight loss compared to vehicle control-treated animals at the termination of the study (*p* = 0.009) (Fig. 1c, d).

### TAK1 inhibition with takinib reduces overall joint inflammation and cartilage damage

Histopathologic effects of takinib were evaluated in the joints of disease animals on day 36 of the study. Vehicle disease control animals had histopathologic changes consistent with those seen in type II collagen-induced arthritis in most joints, with scores ranging from minimal to severe. Microscopic alteration included infiltration of synovium and periarticular tissue with neutrophils and mononuclear inflammatory cells (inflammation), marginal zone pannus, bone resorption, and cartilage damage (proteoglycan loss, chondrocyte death, and collagen matrix destruction). We first evaluated the effects of takinib on overall joint pathology including measures taken from forepaws, hind paws, ankles, knees, and wrists of all animals. Takinib reduced overall joint pathology by ~ 30% compared to vehicle control (*p* = 0.02) (Fig. 2a). Furthermore, takinib significantly reduced inflammation (*p* = 0.019) and cartilage damage (*p* = 0.013) with concurrent trends toward reduction of pannus, bone resorption, and periosteal bone scores (Fig. 2b–f). Periosteal bone width was recorded in all joints. Takinib reduced overall periosteal bone width by 35% compared to vehicle control (*p* = 0.06) (Additional file 1: Figure S1).

### Effects of takinib on CIA mice knees

We next explored the effects of takinib on the individual joints of CIA-treated mice. Knees of CIA diseased mice showed varying signs of inflammation, cartilage damage, and pannus and bone resorption consistent with the CIA model (Fig. 3a, b, c,). TAK1 inhibition reduced the overall sum score of knees by ~ 40% (Fig. 3d). Takinib-treated animals significantly reduced the histological score of inflammation and cartilage damage in the knees of treated mice compared to vehicle control (*p* = 0.0044 and *p* = 0.0042, respectively) (Fig. 3e, f). Furthermore, takinib-treated mice showed a trend toward reduced histological scores in pannus and bone resorption (~ 40% each) (Fig. 3g, h).

### Effects of takinib on forepaws and hind paws of diseased animals

**Fig. 4:**
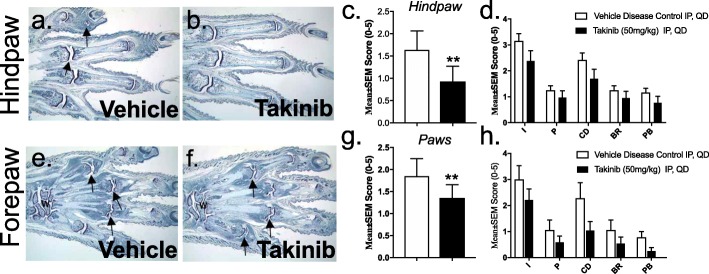
Hind paws from a vehicle control animal have mild inflammation and very minimal cartilage damage in a few digit joints. Arrows identify representative affected joints/regions (**a**). Hind paws from an arthritic animal treated with 50 mg/kg of takinib have very minimal inflammation in the ankle only (**b**). Reduction of inflammation (I), pannus (P), cartilage damage (CD), bone resorption (BR), and periosteal bone formation (PB) were seen in the hind paws of vehicle- and takinib-treated animals (**c**, **d**). Forepaw from a vehicle control animal has severe inflammation and mild cartilage damage with minimal pannus, bone resorption, and periosteal bone formation, in all joints. Arrows identify representative affected joints. “W” identifies wrist (**e**). Forepaw from an arthritic animal treated with 50 mg/kg of takinib has inflammation and cartilage damage with moderate pannus, bone resorption, and periosteal bone formation, in all joints. Arrows identify representative affected joints (**f**). Reduction of inflammation (I), pannus (P), cartilage damage (CD), bone resorption (BR), and periosteal bone formation (PB) were seen in the forepaw of vehicle- and takinib-treated animals (**g**, **h**). All images represent animal with approximate mean summed paw score for the group. *n* = 9–12 ± SEM/vehicle disease control and takinib groups. Two-way ANOVA, Dunnett’s post hoc. *p < 0.05, **p < 0.01, ***p < 0.001

Histological effects of takinib were evaluated in the paws of CIA mice. Hind paws and forepaws of mice were blindly scored for inflammation, cartilage damage, and pannus and bone resorption. Toluidine blue staining showed marked inflammation and cartilage damage with minimal pannus and bone resorption, as well as mild periosteal bone formation, in most joints of diseased animals (Fig. 4a, b, e, f). TAK1 inhibition with takinib reduced the overall sum histological score of hind paws and forepaws compared to vehicle control (*p* = 0.0078 and *p* = 0.0091, respectively) (Fig. 4c, g). Furthermore, trends of reduced clinical scores of inflammation, pannus, cartilage damage, bone resorption, and periosteal bone formation were observed in both hind paws and forepaws of animals with an average 20–30% reduction in disease pathology observed (Fig. 4d, h).

### Pharmacokinetics of takinib

Pharmacokinetics of takinib were evaluated in C3 tagged mice treated with either 50 or 75 mg/kg of takinib i.p. Based on the predicted partition coefficient of takinib (2.56), we expect an absorption of the drug within seconds in the vasculature of the peritoneum. Takinib exhibited rapid plasma clearance as determined by t_½_ of 21 min (Table 1). Takinib showed a one compartment pharmacokinetics as seen by the derivative of Cp over time (Additional file 1: Figure S2). The muscle displayed rapid takinib clearance; however, the spleen revealed delayed takinib clearance with a t_½_ for each tissue at 0.147 and 4.646 h respectively. Furthermore, we show the accumulation of takinib in various other tissues in the mouse, including the heart and tumor (Table 1).

**Table 1:** Takinib pharmacokinetics in C3 tagged mice

Tissue	50 mpk half-life—hours	75 mpk half-life—hours
Plasma	0.2566	0.47
Heart	0.266	0.24
Spleen	4.626	N/A
Tumor	0.3676	0.1657
Muscle	0.147	0.094

Following intraperitoneal (i.p) injections of takinib (50/75 mpk), tissue takinib concentrations were evaluated at 0 h, 1 h, 2 h, 4 h, 8 h, 16 h, and 24 h. T_½_ reported in hours. *Mpk* milligrams per kilogram of body weight

### Anti-inflammatory effects of TAK1 inhibition on RA-FLS cells

**Fig. 5:**
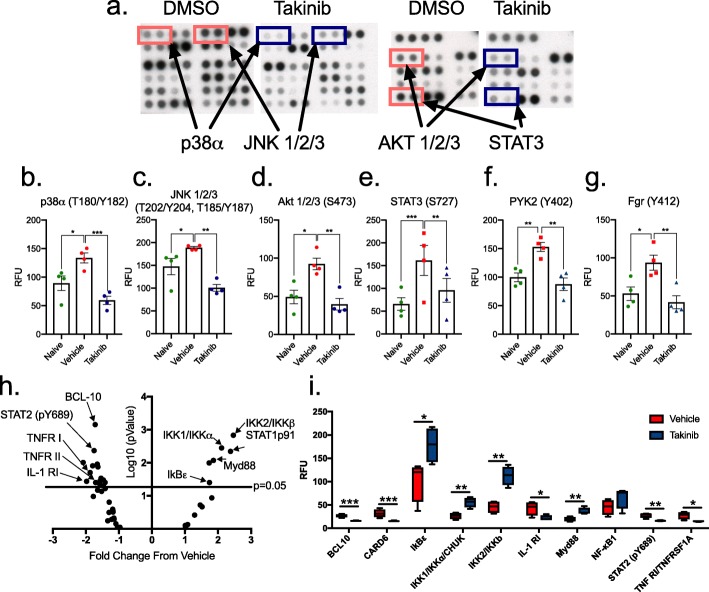
TAK1 inhibition with takinib alters the phosphorylation of kinases and NF-κB protein expression levels in RA-FLS cells. Representative phospho-kinase blots from RA-FLS cells treated with takinib or DMSO and stimulated with TNF (30 ng/mL) for 30 min (**a**). Relative phosphorylation of p38α, JNK1/2/3, Akt 1/2/3, STAT3, PYK2, and Fgr from naïve (un-stimulated *n* = 4 ± SEM, vehicle (TNF + DMSO) *n* = 4 ± SEM, and takinib (TNF + takinib 10 μM) *n* = 4 ± SEM (**b**–**g**) (two-way ANOVA, Dunnett’s post hoc). Volcano plot of NF-κB-associated protein expression 24 h post-TNF (30 ng/mL) stimulation (**h**). Expression of BCL10, CARD6, IkBε, IKK1, IKK2, IL-1R, Myd88, NF-κB1, STAT2, and TNF RI from vehicle- and takinib-treated RA-FLS cells (**i**) vehicle (DMSO) control (*n* = 4 ± SEM), 10 μM takinib (*n* = 4 ± SEM), (Students *t* test). *p < 0.05, **p < 0.01, ***p < 0.001. RFU = relative fluorescent units

TAK1 plays an integral role in cytokine and NF-κB signaling cascades. We hypothesized that TAK1 inhibition of stimulated RA-FLS cells would reduce inflammatory cytokine molecular pathways. To determine the effects of takinib on phosphorylation of various kinases involved in inflammation and TNF signaling, we treated RA-FLS cells with or without takinib (10 μM) followed by 30 min stimulation with TNF (30 ng/mL). We found that takinib significantly reduced the phosphorylation of 21 human kinases including p38α T180/Y182 (*p* < 0.0001), JNK1/2/3 T202/Y204 (*p* < 0.0001), Akt1/2/3 S473 (*p* < 0.0035), STAT3 S727 (*p* < 0.0002), Pyk2 Y402 (*p* < 0.0002), and Fgr Y412 (*p* < 0.0044) (Fig. 5a–g, Additional file 1: Table S2). We further investigated the downstream effects of takinib on the NF-κB signaling pathway. To this end, we treated RA-FLS cells with or without takinib and stimulated them for 24 h in the presence of TNF (30 ng/mL). Out of 45 human NF-κB signaling proteins profiled, 20 were significantly altered in the presence of takinib, including the IKK family members, IKK1/IKKα (*p* < 0.003), IkBε (*p* < 0.039), IKK2/IKKβ (*p* < 0.001), interleukin receptors IL-1RI (*p* < 0.03), IL-17RA (*p* < 0.03), and Il-18Rα (*p* < 0.03). Furthermore, changes were seen in STAT1p91 (*p* < 0.004), STAT2 pY689 (*p* < 0.004), and the TNF receptors TNFR I (*p* < 0.01) and TNFR II (*p* < 0.04) (Fig. 5h, i, Additional file 1: Table S3).

**Fig. 6:**
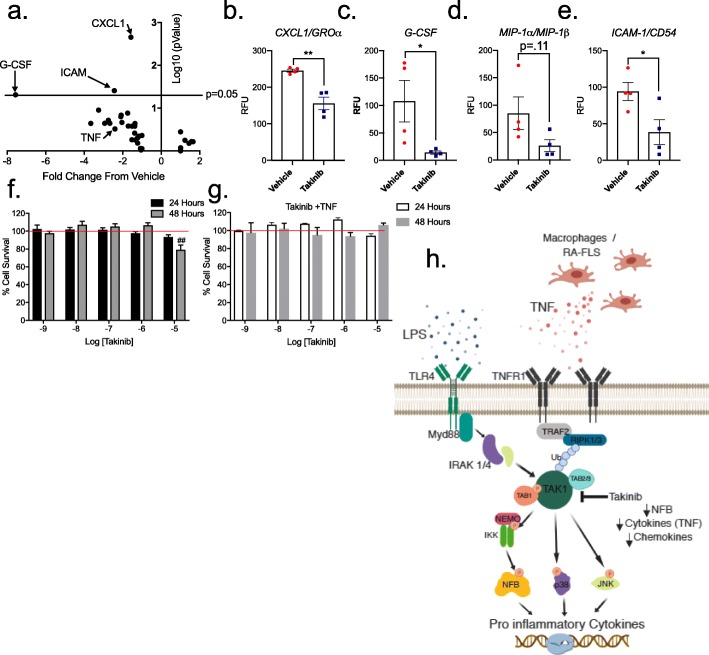
Takinib reduces the cytokine and chemokine response in pro-inflammatory stimulated RA-FLS cells. RA-FLS cells were activated with TNF (30 ng/mL) and treated with 10 μM takinib or DMSO. A total of 40 cytokine and chemokine proteins were profiled. Takinib reduces the expression of G-CSF, ICAM, and CXCL1 (**a**, **b**, **c**, **e**) with modest change in MIP-1α (**d**) compared to vehicle control. Vehicle (DMSO) control (*n* = 4 ± SEM), 10 μM takinib (n = 4 ± SEM) (Students *T* test). RA-FLS cells were plated at 80% confluency and serum starved in 1% FBS media overnight following treatment with takinib at indicated concentrations (**f**) or takinib and TNF (30 ng/mL) (**g**) (*n* = 3 ± SEM) (two-way ANOVA, Dunnett’s post hoc). Relative cell viability was compared to DMSO control-treated cells following 24 and 48 h of treatment. Schematic of TAK1 signaling in pro-inflammatory stimulated cells and downstream effects (**h**). RFU = relative fluorescent units. *p < 0.05, **p < 0.01, ***p < 0.001

Due to TAK1’s tight regulation of TNF signaling, we next sought to test the anti-inflammatory effects of takinib on RA-FLS cells stimulated with exogenous TNF. RA-FLS cells were either treated with or without takinib (10 μM) and stimulated for 24 h with TNF (30 ng/mL). Takinib overall reduced the majority of the pro-inflammatory cytokines profiled including GROα (*p* < 0.002), G-CSF (*p* < 0.04), MIP-1α/MIP-1β (*p* = 0.11), and ICAM (*p* < 0.03) (Fig. 6a–e).

To further test the hypothesis that TAK1 inhibition will disrupt inflammatory cytokine signaling in RA-FLS cells, we stimulated RA-FLS cells with the Tlr4 pathway activator lipopolysaccharide (LPS). Twenty-four hours following LPS (100 ng/mL) stimulation, the cytokine/chemokine profile of either takinib- or vehicle-treated cells were evaluated. Chemokines CCL2 and CXCL1 were found to be significantly downregulated by takinib (*p* < 0.0009 and *p* < 0.0003, respectively). Furthermore, pro-inflammatory cytokines IL-6 and IL-8 were reduced in takinib-treated cells by 33% and 57% (*p* < 0.055 and *p* < 0.006), respectively. No significant changes were seen in MIF and Serpin E1 protein levels (Additional file 1: Figure S3).

Next, we investigated the cytotoxic effects of takinib on RA-FLS cells. Following 24 or 48 h of treatment with takinib, 10 μM takinib treatment induced a significant amount of cell death at 48 h compared to vehicle control (*p* < 0.002) (Fig. 6f). Furthermore, previous studies have shown that in some cell types, TAK1 inhibition in combination with TNF can induce cell apoptosis [28]. Here, we treated RA-FLS cells with takinib at varying doses in conjunction with TNF (30 ng/mL) to investigate the cytotoxicity of takinib treatment in the presence of TNF. Minimal cytotoxicity was seen at 24 and 48 h post-TNF treatment, indicating TAK1-TNF induction of apoptosis does not occur in RA-FLS cells (Fig. 6g). A schematic of TAK1 signaling cascades with downstream effects of takinib inhibition is shown in Fig. 6h.

## Discussion

Here, we show that TAK1 has promise as a therapeutic target for treating RA as seen by the ability of takinib, a TAK1 inhibitor, to reduce clinical score, as well as reduce disease-associated weight loss in CIA mice. Furthermore, we show through histological analysis that takinib treatment reduced inflammation, pannus, cartilage damage, bone resorption, and periosteal bone formation in these animals. Individual joint analysis showed takinib greatly reduced inflammation and cartilage damage in the knees of diseased animals compared to vehicle control. Additional analysis of the forepaws and hind paws exhibited similar trends of takinib reduction of joint scoring categories. Variation seen in joint efficacy may be due to differences in FLS phenotypes in different joints, which have previously been shown in RA patients [29], although further phenotypic studies in response to takinib are needed to verify this hypothesis. Furthermore, the pharmacokinetics of takinib demonstrated a very short serum half-life, suggesting it is rapidly cleared or absorbed throughout the body, as implied by the low-level second-order elimination kinetics observed. Even with limited plasma exposure, takinib reduced disease burden by ~ 40%, supporting the hypothesis that TAK1 may be an advantageous target to treat RA. Future iterations of structure-activity relationship studies of takinib analogs are ongoing and aim at providing longer serum exposure.

Cellular assays using RA-FLS cells showed TAK1 inhibition, with takinib reducing the pro-inflammatory cytokine milieu associated with immune challenge. Although TAK1 plays an essential role in mediating LPS- and TNF-associated pathways, it also has been implicated in mediating numerous cytokine signal transduction pathways such as IL-1 [30, 31]. Due to its high redundancy in pro-inflammatory signal transduction, TAK1 makes an attractive target to mediate not only TNF-dominated diseases, but various other cytokine-mediated diseases. Additionally, although other groups have shown that TNF + TAK1 may induce apoptosis/necrosis in certain cell types, our studies indicate that addition of TNF and takinib showed no apoptosis [32, 33, 28]. This may be supported by shown here showing the downregulation of TNF receptors on RA-FLS cells in the presence of takinib and TNF thus dampening the TNF signaling cascade. Thus, the therapeutic potential of takinib may be expanded outside of just TNF inflammatory diseases. Overall, these studies lay the premise that small molecule therapies targeting TNF production/secretion may provide a novel therapeutic axis in treating RA.

Biologic medications and combinations of disease-modifying drugs have transformed outcomes for patients with chronic autoimmune diseases (e.g., rheumatoid arthritis, Crohn’s or inflammatory bowel disease). With TNF blockers, the key mechanism is prevention of TNF binding to its receptors [34]. TNF has been shown to be a key driver of chronic pain and inflammation and can stimulate its own production locally and also the production of other pro-inflammatory cytokines. Despite the success and efficacy achieved with anti-TNF biologics in a large majority of patients, 20–50% of patients show either no response or, despite an initial response under a maintenance dose, show disease recurrence [14]. Non-responsiveness in patients can be associated with a failure to reduce circulating TNF levels, most commonly due to an immune response to these recombinant protein-based drugs resulting in rapid clearance [35, 36]. Additionally, long-term administration of these biologics is associated with increased risk of infection, malignancy, and other serious adverse events related to immune-sensitization to the drugs themselves [37, 38]. Newer approaches, including JAK inhibitors, are emerging as potential alternates in the treatment of RA [39, 40]. Clearly, increasing our repertoire of available molecularly targeted therapies that can be taken orally will provide an attractive alternate to drugs that target circulating TNF. Based on findings shown herein, we believe that a selective TAK1 inhibitor adds an additional therapeutic approach to reduce the effects of TNF and mitigate RA symptoms and damage.

## Conclusions

These findings indicate the reduction of pro-inflammatory mediators in disease state as well as the reduction of histological tissue damage in takinib-treated animals. Taken together, the data shown here supports the therapeutic potential of TAK1 targeted inhibitors to treat inflammatory arthritis.

## Supplementary information

**Additional file 1: Figure S1.** Takinib reduces periosteal bone width in CIA mice. Mice treated daily with Takinib (50 mg/kg) showed reduced periosteal bone width compared to vehicle treated. *N*=9-12±SEM. *p< 0.05, **p<0.01, ***p<0.001 Student’s T-test. **Figure S2.** Standard Curve of Takinib for LC-MS analysis. Standard curve of Takinib was made in murine plasma for PK analysis of Takinib in vivo. **Figure S3.** Takinib reduces the cytokine and chemokine 1 response in pro-inflammatory stimulated RA-FLS. cells. RA-FLS cells were activated with LPS (10ng/mL) and treated with 10μM Takinib or DMSO. 40 cytokine and chemokine proteins were profiled. Takinib reduces the expression of CCL2, CXCL1, IL-6, and IL-8 (a.-d.), compared to vehicle control. No changes were observed in MIF and Serpin E1 expression levels (e., f.).Vehicle (DMSO) control (*n*=4 ±SEM), 10μM Takinib (n=4 ± SEM), (Two-way ANOVA with Dunnett’s post hoc). **Table S1**. CIA disease mice were evaluated at day 36 for disease progression. Overall area under curve (AUC) is reported and % inhibition from vehicle disease control. **Table S2.** RA-FLS cells had no treatment (naïve) or were stimulated for 30 minutes with TNF, either treated with or without takinib. 45 phospho-kinase proteins were profiled. Mean and standard deviation (SD) are reported for each analyte *n*=4. **Table S3.** RA-FLS cells were stimulated for 24 hours with TNF, either treated with or without takinib. 45 NF-κB associated proteins were profiled. Mean and standard deviation (SD) are reported for each analyte n=4.

## Data Availability

Please contact author for data requests.

## Abbreviations

CFA: Complete Freund’s adjuvant
CIA: Type II collagen-induced arthritis
JAK: Janus kinase
PK: Pharmacokinetics
RA: Rheumatoid arthritis
RA-FLS: Rheumatoid arthritis fibroblast-like synoviocytes
STAT: Signal transducer and activator of transcription proteins
TAK1: Transforming growth factor β-activated protein kinase 1
TNF: Tumor necrosis factor
