# Supplementary material for: Pharmacological inhibition of TAK1, with the selective inhibitor takinib, alleviates clinical manifestation of arthritis in CIA mice
Source: Arthritis Res Ther. 2019 Dec 17;21:292. doi: 10.1186/s13075-019-2073-x (PMC6918687; doi:10.1186/s13075-019-2073-x)
Supplement: Supplementary file 1 — Additional file 1: Figure S1. Takinib reduces periosteal bone width in CIA mice. Mice treated daily with Takinib (50 mg/kg) showed reduced periosteal bone width compared to vehicle treated. N=9-12±SEM. *p< 0.05, **p<0.01, ***p<0.001 Student’s T-test. Figure S2. Standard Curve of Takinib for LC-MS analysis. Standard curve of Takinib was made in murine plasma for PK analysis of Takinib in vivo. Figure S3. Takinib reduces the cytokine and chemokine 1 response in pro-inflammatory stimulated RA-FLS. cells. RA-FLS cells were activated with LPS (10ng/mL) and treated with 10μM Takinib or DMSO. 40 cytokine and chemokine proteins were profiled. Takinib reduces the expression of CCL2, CXCL1, IL-6, and IL-8 (a.-d.), compared to vehicle control. No changes were observed in MIF and Serpin E1 expression levels (e., f.).Vehicle (DMSO) control (n=4 ±SEM), 10μM Takinib (n=4 ± SEM), (Two-way ANOVA with Dunnett’s post hoc). Table S1. CIA disease mice were evaluated at day 36 for disease progression. Overall area under curve (AUC) is reported and % inhibition from vehicle disease control. Table S2. RA-FLS cells had no treatment (naïve) or were stimulated for 30 minutes with TNF, either treated with or without takinib. 45 phospho-kinase proteins were profiled. Mean and standard deviation (SD) are reported for each analyte n=4. Table S3. RA-FLS cells were stimulated for 24 hours with TNF, either treated with or without takinib. 45 NF-κB associated proteins were profiled. Mean and standard deviation (SD) are reported for each analyte n=4. [file 13075_2019_2073_MOESM1_ESM.docx]

**Pharmacological inhibition of TAK1, with the selective inhibitor takinib, alleviates clinical manifestation of arthritis in CIA mice**

Scott A. Scarneo^1^ M.S., M.B.A., Liesl S. Eibschutz M.S.^1^, Phillip J. Bendele^2^ B.A., M.B.A., Kelly W. Yang^1^, Juliane Totzke^1^ Ph.D., Philip Hughes^1^ Ph.D., David A Fox^3^ Ph.D., Timothy A.J. Haystead^1^ Ph.D.

**Supplementary Material**

**Figure S1.** Takinib reduces periosteal bone width in CIA mice. Mice treated daily with Takinib (50mg/kg) showed reduced periosteal bone width compared to vehicle treated. N=9-12±SEM. *p< 0.05, **p<0.01, ***p<0.001 Student’s T-test.

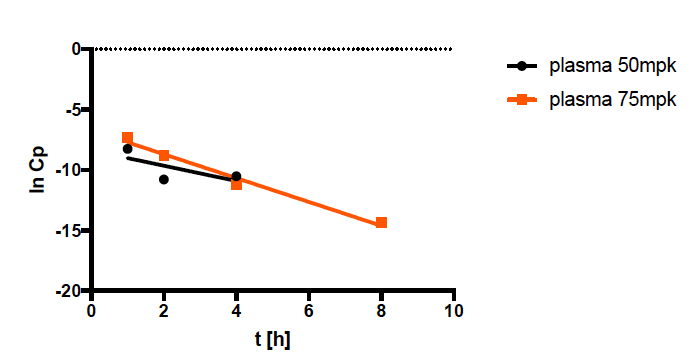


**Figure S2.** Standard Curve of Takinib for LC-MS analysis. Standard curve of Takinib was made in murine plasma for PK analysis of Takinib *in vivo*.

**Figure S3.**

Takinib reduces the cytokine and chemokine 1 response in pro-inflammatory stimulated RA-FLS

cells. RA-FLS cells were activated with LPS (10ng/mL) and treated with 10μM Takinib or DMSO. 40 cytokine and chemokine proteins were profiled. Takinib reduces the expression of CCL2, CXCL1, IL-6, and IL-8 (a.-d.), compared to vehicle control. No changes were observed in MIF and Serpin E1 expression levels (e., f.).Vehicle (DMSO) control (n=4 ±SEM), 10μM Takinib (n=4 ± SEM), (Two-way ANOVA with Dunnett’s post hoc).

| **Group** | **Number of animals developing disease** | **Mean Clinical Arthritic Score (AUC)** | **SE** | **% Inhibition from Vehicle Disease** |
| --- | --- | --- | --- | --- |
| Disease Control | 0% | 0.00 | 0.00 | 100% |
| Vehicle Disease Control | 75% | 21.00 | 2.19 | 0% |
| Takinib Disease Treated | 91.60% | 14.19 | 2.83 | 32% (p=0.08) |

**Table S1**. CIA disease mice were evaluated at day 36 for disease progression. Overall area under curve (AUC) is reported and % inhibition from vehicle disease control.

|  | **Naïve** | | **DMSO** | | **Takinib** | |
| --- | --- | --- | --- | --- | --- | --- |
| **Protein** | **Mean** | **SD** | **Mean** | **SD** | **Mean** | **SD** |
| p38α | 89.391 | 25.74656 | 133.7875 | 17.3224 | 59.40763 | 14.25904 |
| ERK1/2 | 88.67775 | 27.51688 | 118.6624 | 18.97761 | 95.02375 | 20.51589 |
| JNK 1/2/3 | 147.5246 | 36.06355 | 188.7948 | 5.348063 | 100.6526 | 14.85965 |
| GSK-3α/β | 198.9934 | 26.27546 | 230.0453 | 3.857871 | 191.6206 | 20.45042 |
| p53 (pS392) | 67.22875 | 24.50631 | 160.6421 | 16.14043 | 83.6435 | 24.74071 |
| EGF R | 104.2651 | 23.47645 | 141.9319 | 12.523 | 60.58963 | 13.06521 |
| MSK1/2 | 242.2766 | 4.73404 | 246.8179 | 3.731486 | 239.7188 | 6.892202 |
| AMPKα1 | 192.5799 | 21.68947 | 211.1784 | 20.08927 | 111.9564 | 16.83798 |
| Akt 1/2/3 (pS473) | 49.0655 | 17.7872 | 92.621 | 15.194 | 39.51013 | 15.12047 |
| Akt 1/2/3 (pT308) | 164.0194 | 26.60498 | 199.6485 | 13.06341 | 122.7503 | 45.09716 |
| p53 (pS46) | 122.5326 | 37.32683 | 194.032 | 14.74363 | 126.377 | 49.50179 |
| TOR | 168.4626 | 9.434922 | 156.1958 | 21.29862 | 129.6068 | 23.63791 |
| CREB | 227.2879 | 19.77745 | 241.5688 | 9.119509 | 232.3464 | 12.79505 |
| HSP27 | 92.49888 | 18.79752 | 223.3803 | 35.29035 | 202.916 | 56.84297 |
| AMPKα2 | 200.415 | 15.36537 | 209.3104 | 10.26088 | 187.6195 | 19.06983 |
| β-Catenin | 97.775 | 20.18453 | 114.4099 | 24.40617 | 57.16 | 20.47768 |
| p70 S6 Kinase (T389) | 69.03063 | 21.45488 | 140.5273 | 33.36979 | 78.64938 | 38.3002 |
| p53 (pS15) | 37.01838 | 12.97487 | 85.94463 | 22.68054 | 47.47813 | 18.27288 |
| c-Jun | 125.7188 | 39.67621 | 201.299 | 13.20365 | 196.6019 | 43.05628 |
| Src | 187.6703 | 10.14205 | 202.4673 | 14.30016 | 176.3574 | 14.23866 |
| Lyn | 91.49513 | 25.41701 | 119.3705 | 29.2036 | 69.33913 | 20.34469 |
| Lck | 42.6155 | 13.43847 | 79.08113 | 26.95396 | 44.393 | 11.46362 |
| STAT2 | 218.6594 | 14.46681 | 226.2159 | 6.502308 | 200.5311 | 14.62597 |
| STAT5a | 128.8256 | 24.40655 | 128.9481 | 6.621542 | 99.70088 | 15.80837 |
| p70 S6 Kinase | 100.2185 | 34.04018 | 166.1611 | 36.63209 | 110.3643 | 44.4265 |
| RSK1/2/3 | 108.5098 | 36.95201 | 185.329 | 8.192602 | 168.3928 | 25.23193 |
| eNOS | 30.17913 | 9.145844 | 49.96975 | 8.505825 | 41.6005 | 12.50611 |
| Fyn | 108.6744 | 24.68267 | 107.1935 | 29.80033 | 78.89575 | 25.03884 |
| Yes | 165.3398 | 29.29732 | 179.0003 | 21.76658 | 124.3601 | 30.11497 |
| Fgr | 53.07763 | 17.74537 | 93.7105 | 20.00953 | 41.73138 | 17.43388 |
| STAT66 | 171.3735 | 20.74144 | 181.2844 | 21.31855 | 147.8809 | 21.70619 |
| STAT5b | 181.595 | 14.55174 | 182.5331 | 13.63869 | 154.004 | 26.59189 |
| STAT3 (pY705) | 79.54888 | 25.72955 | 112.7603 | 32.99291 | 85.92688 | 34.25089 |
| p27 | 24.83375 | 8.289832 | 73.49075 | 24.86781 | 40.56125 | 13.93223 |
| PLC-γ1 | 38.72975 | 8.123707 | 72.43313 | 9.170878 | 34.4325 | 7.830188 |
| Hck | 155.2668 | 21.89451 | 145.9219 | 29.51875 | 119.1771 | 30.77304 |
| Chk-2 | 170.0696 | 21.48718 | 178.9215 | 22.44123 | 86.85488 | 34.28127 |
| FAK | 180.2579 | 31.81548 | 198.8238 | 35.75799 | 142.7814 | 46.25937 |
| PDGF Rβ | 116.52 | 18.57044 | 133.9915 | 33.01811 | 50.4425 | 10.153 |
| STAT5a/b | 164.7749 | 21.20758 | 180.6776 | 15.23359 | 146.267 | 11.31607 |
| STAT3 (pS727) | 65.49188 | 27.63169 | 161.3745 | 66.19371 | 95.82013 | 52.3799 |
| WNK1 | 174.4721 | 34.76669 | 230.0596 | 10.17078 | 218.9865 | 20.8665 |
| PYK2 | 99.95625 | 15.33222 | 152.9993 | 15.93586 | 87.75188 | 21.70773 |
| PRAS40 | 234.6085 | 13.16464 | 251.0528 | 2.114253 | 244.5596 | 8.685761 |
| HSP60 | 18.93563 | 1.656054 | 59.9635 | 30.02242 | 40.313 | 13.77095 |

**Table S2.**

RA-FLS cells had no treatment (naïve) or were stimulated for 30 minutes with TNF, either treated with or without takinib. 45 phospho-kinase proteins were profiled. Mean and standard deviation (SD) are reported for each analyte n=4.

|  | **DMSO** | | **Takinib** | |
| --- | --- | --- | --- | --- |
| **Protein** | **Mean** | **SD** | **Mean** | **SD** |
| ASC | 38.51213 | 8.190283 | 29.77338 | 6.99303 |
| BCL10 | 27.10275 | 3.43087 | 15.75775 | 0.953151 |
| CARD6 | 31.83913 | 8.801112 | 15.28838 | 1.220783 |
| CD40/TNFRSFS | 42.49488 | 18.43122 | 23.956 | 5.96586 |
| clAP1/BIRC2 | 41.89263 | 14.47695 | 25.46725 | 2.762223 |
| clAP2/BIRC3 | 26.65875 | 5.965177 | 18.81563 | 3.213953 |
| FADD/MORT1 | 39.8005 | 13.50073 | 36.44838 | 9.567851 |
| Fas/TNFRSF6/CD95 | 168.342 | 51.50029 | 172.1241 | 38.64513 |
| IkBα | 40.69913 | 18.83542 | 33.40438 | 9.406067 |
| IkBε | 102.5876 | 44.19701 | 178.592 | 37.69026 |
| IKK1/IKKα/CHUK | 26.43413 | 6.784912 | 55.68088 | 10.65121 |
| IKK2/IKKβ | 45.66263 | 13.02529 | 112.6481 | 20.40636 |
| IKKγ/NEMO | 111.6461 | 49.95772 | 107.307 | 34.54756 |
| IL-1 RI | 42.90875 | 14.70256 | 21.67638 | 5.916425 |
| IL-17 RA | 32.71325 | 8.126371 | 19.1835 | 6.379366 |
| IL-18 Rα | 27.674 | 7.245976 | 17.5855 | 1.743287 |
| IRAK1 | 30.8795 | 9.586713 | 26.78075 | 4.54388 |
| IRF5 | 24.16513 | 5.557846 | 16.411 | 0.953633 |
| IRF8 | 24.457 | 5.3462 | 17.251 | 1.278453 |
| JNK1/2 | 23.304 | 5.105037 | 19.75688 | 3.868086 |
| JNK2 | 20.43013 | 4.815083 | 17.3675 | 2.418493 |
| LTBR/TNFRSF3 | 25.37325 | 8.630349 | 22.136 | 3.115312 |
| Metadherin/AEG-1 | 17.89363 | 3.504259 | 31.01175 | 6.163681 |
| MYD88 | 19.47438 | 4.684816 | 36.27613 | 7.372708 |
| NFκB1 | 45.28225 | 16.42038 | 66.143 | 22.94729 |
| NFκB2 | 105.6186 | 52.18158 | 136.3483 | 38.66438 |
| NGF R/TNFRSF16 | 29.93463 | 7.338607 | 16.68163 | 1.556232 |
| p53 | 27.54388 | 6.588111 | 17.84763 | 1.749036 |
| p53 (pS46) | 24.7435 | 5.258391 | 14.93563 | 1.259984 |
| RelA/p65 | 30.79363 | 10.71041 | 55.81425 | 25.0245 |
| RelA/p65 (pS529) | 40.18825 | 17.06219 | 41.56713 | 11.56452 |
| c-Rel | 24.30925 | 6.083116 | 16.51863 | 1.887147 |
| SHARPIN | 20.29138 | 4.663164 | 17.05638 | 2.117888 |
| SOCS6 | 67.31375 | 29.60883 | 64.37413 | 26.31954 |
| STAT1p91 | 48.85475 | 15.02932 | 116.4024 | 26.66525 |
| STAT1 (pY701) | 15.3465 | 0.918876 | 15.5275 | 0.884003 |
| STAT2 | 97.29513 | 65.95092 | 148.6364 | 33.32185 |
| STAT2 (pY689) | 27.93613 | 5.289005 | 15.96013 | 0.906365 |
| STING/TMEM173 | 98.30363 | 34.40654 | 106.8409 | 34.87692 |
| TLR2 | 25.723 | 5.692067 | 15.725 | 0.912449 |
| TNF RI/TNFRSF1A | 27.51125 | 8.064065 | 14.70338 | 0.919697 |
| TNF RII/TNFRSF1B | 28.79925 | 7.943355 | 18.3335 | 2.63936 |
| TRAF2 | 27.25638 | 8.470667 | 17.884 | 1.727819 |
| TRAIL R1/DR4 | 28.07313 | 10.69258 | 21.60238 | 5.169506 |
| TRAIL R2/DR5 | 122.255 | 88.1125 | 91.34088 | 53.56275 |

**Table S3.**

RA-FLS cells were stimulated for 24 hours with TNF, either treated with or without takinib. 45 NF-κB associated proteins were profiled. Mean and standard deviation (SD) are reported for each analyte n=4.
